# Preparation and Characterization of Transethosome Formulation for the Enhanced Delivery of Sinapic Acid

**DOI:** 10.3390/pharmaceutics15102391

**Published:** 2023-09-27

**Authors:** Yousef A. Bin Jardan, Abdul Ahad, Mohammad Raish, Fahad I. Al-Jenoobi

**Affiliations:** Department of Pharmaceutics, College of Pharmacy, King Saud University, Riyadh 11451, Saudi Arabia

**Keywords:** stratum corneum, lipid-based vesicles, ultra-deformable vesicles, dermal/transdermal

## Abstract

Sinapic acid (SA) is a bioactive phenolic acid; its diverse properties are its anti-inflammatory, antioxidant, anticancer, and antibacterial activities. The bioactive compound SA is poorly soluble in water. Our goal was to formulate SA-transethosomes using thin-film hydration. The prepared formulations were examined for various parameters. In addition, the optimized formulation was evaluated for surface morphology, in-vitro penetration studies across the Strat M^®^, and its antioxidant activity. The optimized formulation (F5) exhibited 74.36% entrapment efficacy. The vesicle size, zeta potential, and polydispersity index were found to be 111.67 nm, −7.253 mV, and 0.240, respectively. The surface morphology showed smooth and spherical vesicles of SA-transethosomes. In addition, the prepared SA-transethosomes exhibited enhanced antioxidant activity. The SA-transethosomes demonstrated considerably greater penetration across the Strat M^®^ membrane during the study. The flux of SA and SA-transethosomes through the Strat M^®^ membrane was 1.03 ± 0.07 µg/cm^2^/h and 2.93 ± 0.16 µg/cm^2^/h. The enhancement ratio of SA-transethosomes was 2.86 ± 0.35 compared to the control. The SA-transethosomes are flexible nano-sized vesicles and are able to penetrate the entrapped drug in a higher concentration. Hence, it was concluded that SA-transethosome-based approaches have the potential to be useful for accentuating the penetrability of SA across the skin.

## 1. Introduction

There has been a long-term focus on various approaches adopted for the dermal/transdermal delivery of numerous actives. Amongst the various overtures, phospholipid-based drug delivery systems are an attractive choice for the dermal/transdermal delivery of various pharmaceutical agents [1].

Researchers have uncovered a variety of strategies for bypassing the stratum corneum barrier to deliver drugs effectively to the skin. In 2015, the triamcinolone liposomal dermal delivery system was developed as the first such approach [2]. Because liposomes are rigid, they fail to penetrate deep into the skin and accumulate in the superficial layer [3]. Cevc and Blume overcame liposome rigidity with the development of deformable liposomes [4]. Along with phospholipids, deformable liposomes consist of edge activator(s) and water, which destabilize the lipid bilayers; this increases the elasticity of vesicles. This formulation concept allows deeper drug penetration into the skin [5,6,7,8]. In another study, Touitou et al. investigated elastic vesicular systems called ethosomes. Ethosomes are distinguished from liposomes by the presence of ethanol, phospholipids, and water [9]. Since ethanol fluidizes lipid membranes, it makes vesicles more flexible by interfering with the lipid bond between the skin and vesicles. As a result of this ethanol property, drugs entrapped in ethosomes can pass more easily through stratum corneum barriers [8,10,11]. Transethosomes are a more advanced vesicle delivery system that contains both an edge activator and ethanol. Transethosomes offer the advantages of both ethosomes and deformable liposomes. In addition, transethosomes exhibit improved penetration and deposition properties [12,13]. Several studies have previously attempted to enhance the dermal/transdermal delivery of various drugs using transethosomes [13,14,15,16,17]. Previously, lipid-based nano-vesicles were prepared for the delivery of ferulic acid, which is also a phenolic acid. It was observed that ferulic acid vesicle formulation demonstrated considerably high permeation across the skin. The authors concluded that ferulic acid may be delivered through or into the skin through vesicular carriers [18]. In another study, caffeic acid ethosomes formulations were prepared for transdermal delivery. Caffeic acid also belongs to the phenolic acid group. The authors concluded that the caffeic acid ethosomes system demonstrated extended drug stability. The prepared ethosomes showed a higher level of caffeic acid permeation and antioxidant activity than the control formulation [19].

Sinapic acid (SA) is a phytochemical that occurs in a wide variety of plants, including spices, citrus fruits, berries, and vegetables [20], as well as oilseeds and cereals [21,22]. Studies have reported positive results for SA against several diseases, for instance cancer [23], diabetes [24,25,26,27,28], anxiety [29], inflammation [30], infections [31], neurodegeneration [32], and oxidative stress [33]. SA is a poorly soluble bioactive compound in water and has a demonstrated low in-vitro dissolution rate, which could lead to poor oral bioavailability [34]. Several studies have shown that flavonoids including SA are potent antioxidants that defend the body against oxidative stress and free radicals. SA may also counteract chemical-induced toxic reactions. In antecedent studies, SA-cyclodextrin inclusion complexes prepared by two different techniques demonstrated ameliorated antioxidant properties. The authors indicated that this is possibly as a result of increasing SA solubility in complexes [35,36]. SA and its derivatives are valuable in the food, cosmetic, and pharmaceutical industries [36]. Due to a lack of research available on the formulation development of SA systems for dermal/transdermal delivery, it appears that the SA-transethosomes may be of interest. In the present investigation, phospholipid 90 G (PL90), sodium deoxycholate (SDC), and ethanol were combined in various ratios to produce SA-transethosomes. The prepared SA-transethosomes vesicles were assessed for several parameters. Moreover, the best formulation was examined for its in-vitro permeation and antioxidant activity.

## 2. Materials and Methods

Methanol was sourced from “BDH, England”, and chloroform was acquired from “Sigma-Aldrich, St. Louis, MI, USA”. The Phospholipon 90 G was supplied by “Phospholipid GmbH Nattermannallee, Germany”. The SDC and SA were bought from “AppliChem Panreac, Darmstadt, Germany” and “Carbosynth limited, Berkshire, UK” respectively. Milli-Q water was obtained from “Milipore, Molsheim, Cedex, France”.

### 2.1. Preparation of SA-Transethosomes

A “thin-film hydration” approach was used to formulate SA-transethosomes containing varying PL90/SDC ratios (Table 1). 

In short, the SA-transethosomes were formulated by solubilizing the SA (10 mg), PL90, and SDC in 10 mL methanol: chloroform (1:2, *v*/*v*) and transferring to a flask with a round bottom. The organic phase was eliminated via the rotary evaporation process (HS-2005S, HahnShin Scientific, Bucheon, Republic of Korea); as a result, a thin, dry lipid film was formed. A 10 mL phosphate-buffered saline solution containing ethanol was then used to rehydrate the film after the organic phase was removed completely. After the hydration process, a dispersion of lipid mixture was produced; this coarse dispersion was sonicated for 5 min with a gap of 2 min and 50 amplitudes at 4 °C to produce SA-transethosomes [7,37]. As a final step, the formulations were stored in a refrigerator and further evaluated for different aspects. 

### 2.2. Analysis of the Vesicles Sizes, and Polydispersity Index (PDI) of SA-Transethosomes

A “Zetasizer Nano ZS” was utilized to determine vesicle size using the dynamic light scattering technique at 25 °C. During preparation for analysis, samples were diluted 100 times in Milli-Q water [38]. Parameters such as the PDI are used to describe the uniform size distribution of vesicles for efficient and stable formulations. The PDI and hydrodynamic diameter as Z-average were acquired from the autocorrelation fit of the data.

### 2.3. Assessment of Zeta Potential of SA-Transethosomes

The surface charge of the vesicles is useful for preventing vesicle aggregation and improving the formulation stability. The samples for zeta potential analysis were prepared by diluting the samples with Milli-Q water and evaluated using the Malvern Zetasizer to determine their zeta potential [39]. 

### 2.4. Assessment of Entrapment Efficiency (EE) of SA-Transethosomes

A centrifugation process was carried out to estimate the EE% of SA within the transethosomes. In a cooling centrifuge, a 3 h centrifugation at 15,000 rpm was performed on the samples [40]. The EE% is calculated by comparing the amount of SA added to the transethosomes formulation with the amount remaining in the aqueous phase after centrifuge. We carefully collected the supernatant from the samples and analyzed it using an UV spectrophotometer to estimate the SA content at 322 nm [41]. A formula for calculating EE% is shown below:

$$\text{EE}\%=\left[\frac{\left(\text{Total SA}\;-\;\text{SA detected in supernatant}\right)}{\text{Total SA}}\right]\;\times \;100$$

### 2.5. Surface Morphology of SA-Transethosomes

An investigation of the SA-transethosome surface morphology was undertaken using a transmission electron microscope (TEM). Following the preparation of the sample, a drop of the sample was carefully positioned on a clean copper grid, phosphotungstic acid (1%) was then applied, and the sample was then air-dried. As a final step, the grid was examined under a TEM to determine vesicle shape [3,42].

### 2.6. Assessment of Antioxidant Activity of SA-Transethosomes

In this study, the antioxidant activity of SA-transethosomes was determined using the “2,2-diphenylpicrylhydrazyl (DPPH) assay”. Pure SA (control) and SA-transethosomes were diluted to obtain different concentrations between 0 and 100 µg/mL. 

A spectrophotometer was operated to detect the resultant absorbance at 517 nm [43]. Based on the equation below, the DPPH free radical scavenging by samples was calculated. Further, the SA-transethosomes’ radical scavenging activity against the “ABTS (2,2′-azino-bis(3-ethylbenzothiazoline-6-sulfonic acid)” radical cation was evaluated [44]. In this study, two separate solutions of 2.45 nmol/L potassium persulfate and 7 mmol/L ABTS were prepared in water separately. After mixing the two solutions 1:1, they were kept in the dark at room temperature for six hours. The ABTS radical was produced during this time period. Later, the diluted ABTS radical cation solution was mixed with SA-transethosomes and kept aside the sample for 20 min. After 20 min of incubation at 30 °C, the reaction was measured for absorbance at 734 nm. Test samples were assessed according to the following equation for their ability to quench the ABTS free radicals.

$$\text{Radical Scavenging}\;(\%)\;=\;\frac{\left(\text{Ac}\;-\;\text{As}\right)}{\text{Ac}}\;\times \;100$$

### 2.7. In-Vitro Penetration Study

This in-vitro study was completed via a Strat-M^®^ membrane (Merck Millipore Ltd. Carrigtwohill, Ireland). The pure SA and SA-transethosomes (1 mL) were transferred to the donor cell of the fabricated Franz diffusion cell. The receptor vehicle (85% phosphate buffer pH 7.4 + 15% ethanol) was maintained at 32 °C and continuously stirred. The receiver vehicle (3 mL) was collected and replaced with the fresh vehicle at each time point. A UV spectrophotometer was operated to quantify the samples at 322 nm (Shimadzu 1601 PC, Kyoto, Japan). The absorbance was recorded, and the permeation parameters were calculated.

### 2.8. Statistical Analysis

Statistical analysis was done by unpaired *t*-test using GraphPad InStat^®^, GraphPad Software, Inc., San Diego, CA, USA. * *p* < 0.05 was considered as significant.

## 3. Result and Discussion

In order to enhance the skin’s permeability, drug formulations based on lipid-based vesicles are being investigated. In addition, lipophilic as well as hydrophilic drug(s) could be delivered through lipid-based vesicle formulations [45]. The advantage of these nano-sized formulations is that the entrapped drug(s) can penetrate deeper into the skin by interacting with these lipid vesicles [46]. A variety of vesicular carriers have been developed by investigators as a novel means for delivering actives across the skin [1,47]. Antecedently, drug(s) penetrability across the skin was improved with the advent of liposomes as a delivery system. A liposome consists of phospholipids and cholesterol in a major portion. It was reported that the topical delivery of actives is found to be better via liposomes [48]. Later, it was found that liposomes’ rigid nature limits drug penetration via the skin [49]. To improve drug delivery via the skin route, next-generation nano-sized vesicle carriers are being investigated. During the last few years, researchers have studied nano-sized vesicles such as transfersomes and ethosomes for dermal/transdermal drug delivery. Transferosomes are flexible vesicles and the presence of a phospholipid bilayer and edge activator in the framework helps to promote drug permeation through the skin [50,51,52]. The phospholipid bilayer is destabilized and becomes more flexible with the help of edge activators. A topical application can be made more effective with the use of phospholipids, as they are well accepted by the skin as well as compatible with the skin [53,54]. Hence, transferosomes are an excellent alternative to other delivery systems for delivering therapeutics through the skin. Investigators have further tested additional ways of increasing drug permeation through the skin utilizing ethosomes. Ethosomes are composed of phospholipids, water, and ethanol. For the preparation of ethosomes carriers, ethanol range from 10% to 40% is typically employed [10,11,55,56]. It has been shown in studies that ethosomes have no substantial irritation to skin [57]. Ethosomes contain ethanol, which accentuates permeation of drug and ensures that actives reach well into the epidermis. The mode of action of ethanol mentioned in the literature is that ethanol contributes to the skin’s fluidity by modulating the multiple layers of lipids. In turn, this allows the structure to be flexible and penetrate the skin deeper [58]. In transethosomes, ethanol and edge activators are intermingled to produce the upgraded forms of ethosomes and transfersomes. The ethanol and edge activator impact makes transethosomes more deformable and elastic, enabling drugs to penetrate deeper into the skin. Transethosomes exhibit the advantageous features of both transfersomes and ethosomes. Due to the presence of edge activator and ethanol in the transethosomes, these vesicles demonstrated greater potential in terms of drug penetration [59,60]. 

In this study, thin-film hydration was used in the formulation of SA-transethosomes. As the vesicles’ physical characteristics, such as vesicles size, are also influenced by the pH of the hydrating vehicle, hence the rehydration of the lipid film with phosphate buffered saline was carried, as it has been reported that pH should be close to physiological levels in hydration media [61]. SA-transethosomes vesicles size, EE%, zeta potential, and PDI have been predominantly influenced by phospholipid to SDC ratio and ethanol. It was reported that rehydrating the dried lipid films in ethanolic phosphate buffer saline led to the formation of spontaneous lipid vesicles with micron-sized vesicles with a broad distribution of vesicles sizes [62]. It is likely that these dispersions are multilamellar, similar to other conventional liposomes produced by similar means of film dispersion [61,63]. As a result of sonication, the prepared lipid based vesicles were reduced in size considerably and producing small unilamellar structures [64]. Finally after, sonication semi-transparent SA-transethosomes vesicles were obtained [65].

### 3.1. Vesicles Size and PDI

The Table 1 shows the composition of the SA-transethosomes formulations. It has been reported that vesicles size influences the activity of lipid-based formulations both in vitro and in vivo [65]. Our study therefore investigated the influence of PL90/SDC ratio and ethanol on the vesicles size of SA-transethosomes. 

**Figure 1:**
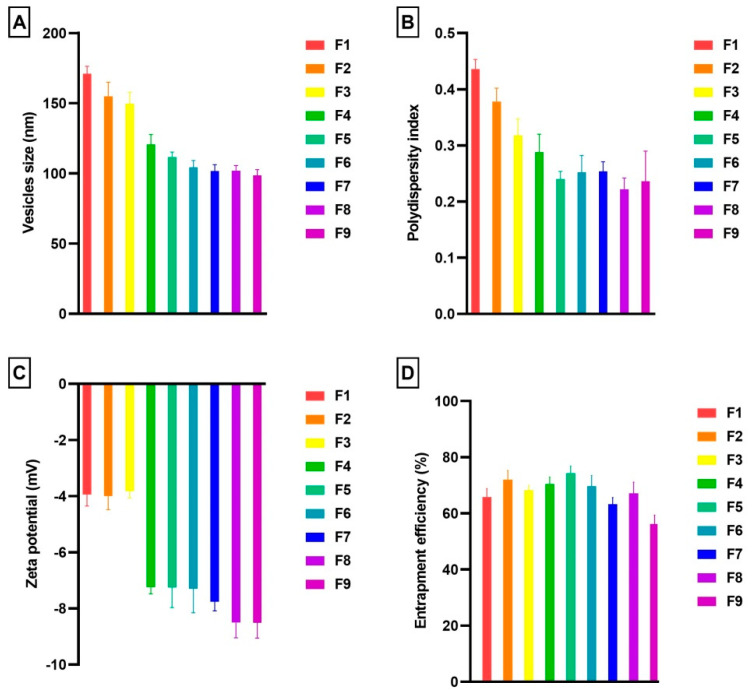
(**A**) Vesicle size, (**B**) polydispersity index, (**C**) zeta potential, and (**D**) entrapment efficiency of prepared SA-transethosomes.

The optimal concentration of ethanol regulates vesicle size by reducing their size and increasing their flexibility and deformability [14,15,16,59,60,66,67,68,69]. In general, it is more effective for the drug to penetrate the skin when the vesicles are smaller in size. In transethosomes vesicles’ shape and size could be regulated and modified by ethanol and an edge activator [70]. As shown in Figure 1, the PL90/SDC ratio pointedly affects the vesicles size, and distribution of vesicles.

In this study, SA-transethosomes demonstrated vesicles sizes ranging from 98.67 ± 4.04 nm to 171.00 ± 5.29 nm (Figure 1A). The average vesicles size was determined to be 123.85 ± 5.68 nm across all prepared nine formulations. It was noted that an enhancement in SDC ratio in the bilayers of transethosomes formulation was correlated with a noteworthy diminution in vesicles size. Further, the size of the SA-transethosomes decreased substantially on increasing the ratio of ethanol. Possibly, this event is influenced by the association among the lipid bilayers and ethanol. Investigators reported that increased ethanol levels result in interpenetration of the Lecithin hydrocarbon chain. As a result, the membrane thickness of transethosomes vesicles decreases and consequently the average vesicles size also decreased [71]. As shown in Fig 1A, the formulation (F1, ethanol 20%) has a vesicles size of 171.00 ± 5.29 nm, but when the ethanol percentage increased to 40%, the vesicles size decreased to 149.67 ± 8.14 nm (F3). Our results are colloborated with previous literature, in a previous study, Salem et al. reported that raising the ethanol level from 10% to 30% caused in a reduction in the average diameter of the vesicles [72]. Further, in another study demonstrated that there is an indirect relationship between the size of the vesicles and the ethanol concentration [73]. Authors suggested that enhancing ethanol concentration reduced phospholipid main transition temperatures. This contributed to partial fluidization of the transethosomes vesicles and the development of nano-sized range vesicles [73]. In another study, it was reported that ethanol levels over 40% were not investigated since elevated ethanol contents resulted in the failure of vesicle formation or the breakdown of already-formed vesicles [74]. A narrow size distribution was observed for all nine prepared formulations (Figure 1B). It was found that only one formulation achieved the PDI value of 0.4, and no formulation had a PDI value greater than 0.5. The obtained values are acceptable and indicate a relatively homogenous vesicular size distribution. Size homogeneity and narrow size distribution are confirmed by values below 0.3. It was reported that a PDI higher than 0.5 is unacceptably high and reflects diverse, heterogeneous vesicle size distributions [75].

### 3.2. Zeta Potential

The vesicles are electrostatically repellent to each other, depending on the degree of repulsion. SA-transethosomes demonstrated the zeta potential ranges from −3.827 ± 0.238 mV to −8.513 ± 0.544 mV (Figure 1C). The average zeta potential was calculated as −6.482 ± 0.483 across all nine prepared formulations. The zeta potentials of the prepared SA-transethosomes demonstrated the increased electrostatic repulsion and stability of transethosomes vesicles. SA-transethosomes (F1–F3) containing a higher ratio of PL90 exhibited a lower negative zeta potential compared to formulations (F7–F9) with lesser PL90 content. In addition, a higher negative zeta potential was observed for formulations with a higher content of ethanol; for instance, formulation F7 (ethanol 20%) showed a negative zeta potential of −7.762, while formulation F9, with a higher content of ethanol (40%), demonstrated a negative zeta potential of −8.513. Almost all of this change might be related to the existence of a higher level of ethanol in the composition. Ogiso et al. cited the high ethanolic content of these nano-size-range vesicles as a major reason for their negative zeta potential. Ethanol contributes negative charges to the phospholipid polar head groups, which ultimately results in an electrostatic repulsion [76].

### 3.3. Entrapment Efficiency

As an assessment of the amount of SA in the transethosomes, the EE% of the prepared formulations was calculated and presented in Figure 1D. The SA-transethosomes demonstrated an EE% ranging from 56% to 74 (Figure 1D). The enhancement of SA solubility in the lipid bilayer in the presence of a surfactant and ethanol could be responsible for its higher entrapment. Variations in EE% may be caused by the PL90, SDC, and ethanol ratio. It was observed that the EE increased up to 30% ethanol. There is a possibility that this is caused by the co-solvent activity of ethanol that enhances SA solubility in transethosomes’ polar phases. As a result, nano-sized vesicles are able to contain an additional amount of the active. Other scenarios could be that the solubilization property of ethanol improves the flexibility of transethosome vesicles, which results in a greater encapsulation of the drug [77]. A decrease in EE% was observed as the ethanol content in the formulations reached 40%. There was a decrease in the EE of SA in vesicles at 40% ethanol, possibly due to vesicle leakage due to higher ethanol ratios [3,5,6,42,78]. In formulations F7 to F9, the EE% was reduced to some extent. It can be concluded that the lower content of PL90 and a higher percentage of ethanol content caused the leakage of the entrapped SA from prepared transethosomes. It was also noted that the EE% decreased with increased SDC concentration. The reason for this is that the edge activator used for the preparation of formulations might be more efficiently incorporated into the PL90 membrane, resulting in a more permeable membrane, thus reducing the EE%. A lower EE% can also be accounted for by the generation of mixed micelles when edge activators are added at higher concentrations [6,79,80].

**Figure 2:**
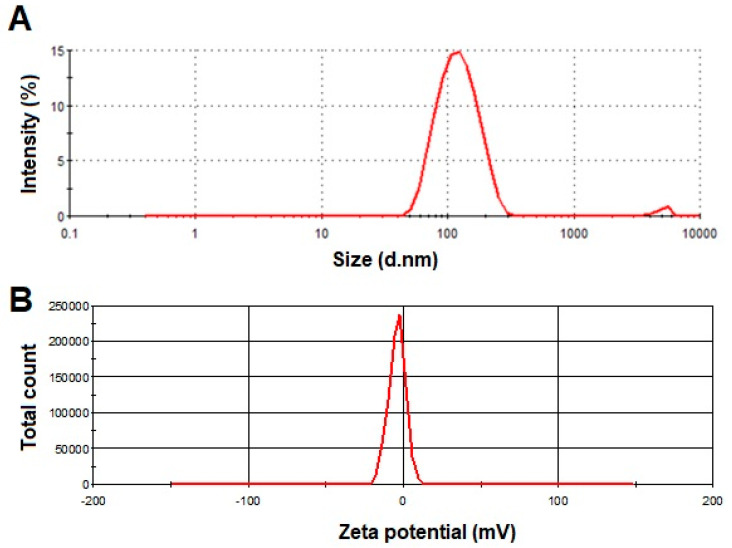
Graph of (**A**) vesicle size and (**B**) zeta potential of optimized SA-transethosomes (F5) formulation.

As demonstrated in Figure 1, formulation F5 was selected as the optimized formulation because it exhibited a maximum EE% of 74.36. The vesicle size and zeta potential of F5 were noted as 111.67 nm and −7.253 mV, respectively (Figure 1 and Figure 2A,B). The homogenous vesicle size distribution of formulation F5 was noted as PDI 0.240 (Figure 1).

### 3.4. Transmission Electron Microscopy

The selected SA-transethosome (F5) formulation was evaluated for surface morphology using the TEM technique. 

**Figure 3:**
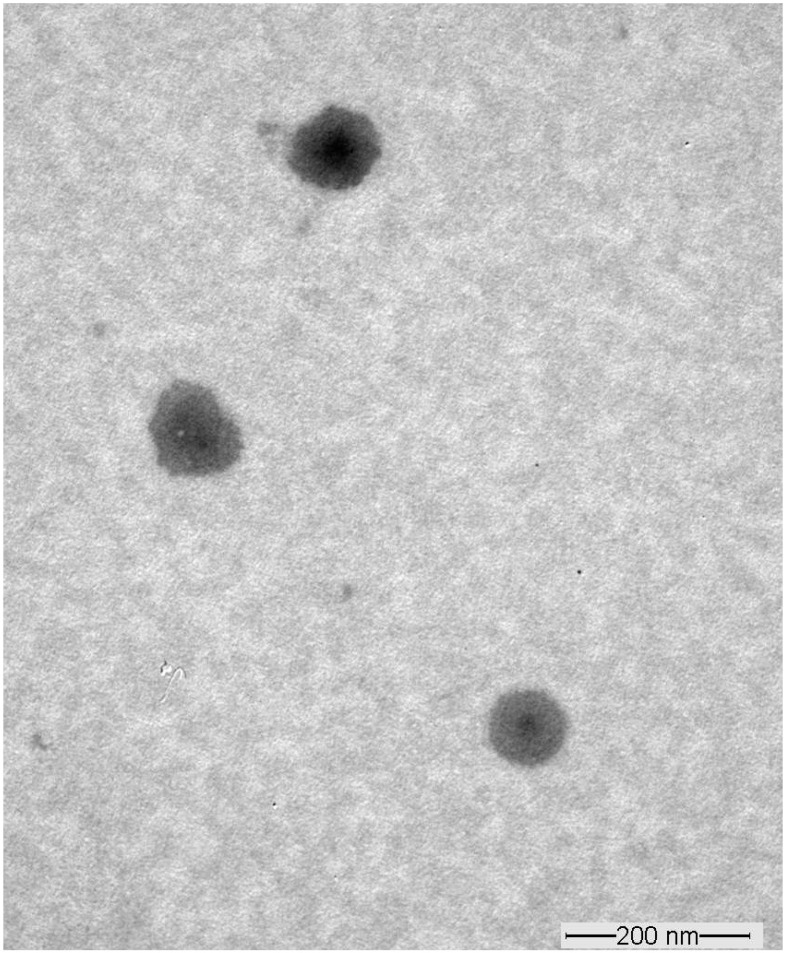
TEM image of optimized SA-transethosomes (F5) formulation.

The TEM examination of vesicles revealed that the outer surface of vesicles was smooth, and there was no indication of an aggregated spherical structure in the SA-transethosomes (Figure 3). Figure 3 shows nanospherical SA-transethosomes of comparable size with no discernible differences. 

### 3.5. Antioxidant Activity

In the present experiment, a DPPH scavenging assay and an ABTS scavenging assay were used to measure antioxidant activity. 

**Figure 4:**
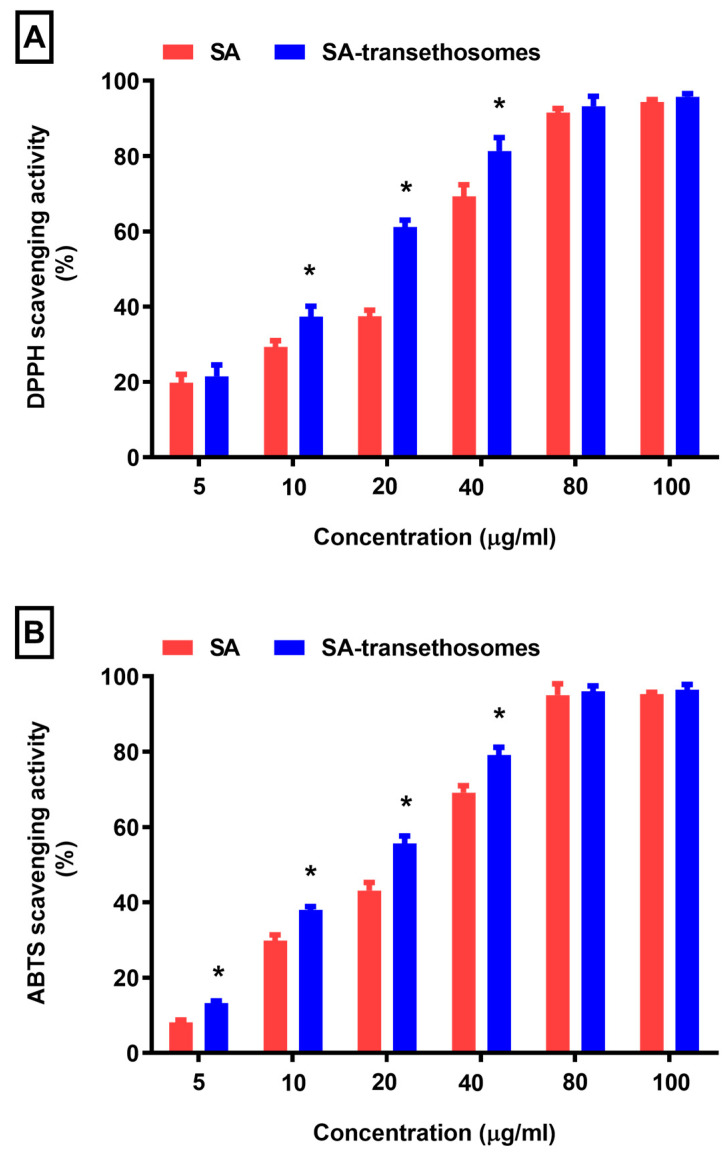
(**A**) DPPH scavenging activity and (**B**) ABTS scavenging activity of optimized SA-transethosomes (F5) formulation (* *p* < 0.05).

Figure 4 show that the SA-transethosomes group displayed a distinct difference in activity. The DPPH radical scavenging potential of pure SA was assessed as 29%, 37%, and 69% at concentrations of 10, 20, and 40 µg/mL. However, the SA-transethosomes presented significantly (*p* < 0.05) better DPPH radical scavenging activity of 37%, 61%, and 81% at concentrations of 10, 20, and 40 µg/mL. Furthermore, there was a considerable difference between the pure SA sample and the SA-transethosomes sample in terms of ABTS scavenging activity. It was found that SA-transethosomes exhibited a significantly improved ABTS scavenging activity (*p* < 0.05) of 13%, 38%, 55%, and 79% at 5, 10, 20, and 40 µg/mL concentrations, compared to pure SA, which demonstrated 8%, 29%, 43%, and 69% ABTS scavenging activity. Hence, in comparison with pure SA, formulation F5 demonstrated improved antioxidant properties. The improved antioxidant of formulation F5 could be due to the presence of excipients that ameliorate the solubility of SA. It was observed that the antioxidant of SA was not changed even after being entrapped in transethosomes vesicles. In addition, it was concluded that the SA-transethosomes exhibited enhanced antioxidant activity compared to pure SA. 

### 3.6. In-Vitro Penetration Study

In this study, the penetration ability of SA-transethosomes across the Strat M^®^ membrane was investigated. 

**Figure 5:**
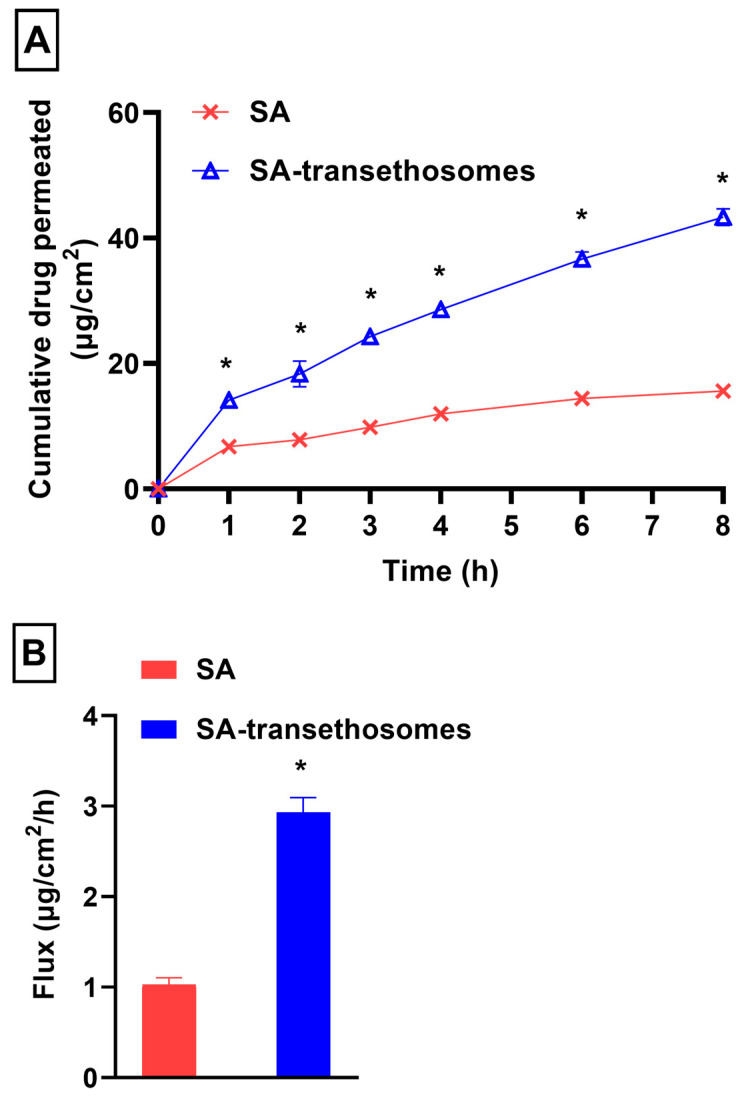
(**A**) Cumulative drug permeated verses time profile and (**B**) flux of optimized SA-transethosomes (F5) formulation (* *p* < 0.05).

The cumulative drug permeated versus time profile of SA and SA-transethosomes is presented in Figure 5A. Transethosomes have been reported to improve drug penetration due to push and pull actions on the stratum corneum’s intercellular interface. The push reaction is the thermodynamic reaction caused by the evaporation of ethanol. Pull impacts are caused by ethanol fluidizing stratum corneum lipids, allowing for new penetration pathways [81]. In this study, the flux of SA across Strat M^®^ was noted as 1.03 ± 0.07 µg/cm^2^/h. However, the flux of SA-transethosomes was significantly (*p* < 0.05) improved, at 2.93 ± 0.16 µg/cm^2^/h (Figure 5B). The enhancement ratio of SA-transethosomes was found to be 2.86 ± 0.35 as compared to control. In terms of drug penetration, SA-transethosomes demonstrated significantly (*p* < 0.05) greater penetration across the Strat M^®^ membrane during the course of the study (Figure 5A). The higher level of drug penetration is possibly a result of their nano-sized vesicles and the flexibility of the prepared SA-transethosomes, which contributes to the higher drug penetration. It is possible that the ethanol present in the transethosome nanovesicles is responsible for this behavior. It increases transethosome penetration power by imparting flexibility and enhancing the penetration [59]. Furthermore, the surfactant and ethanol existent in SA-transethosomes contribute to the diffusivity of SA into the membrane. Both are reported as penetration enhancers in various reports [82,83,84]. 

## 4. Conclusions

SA-transethosomes were formulated using thin-film hydration. As demonstrated in Figure 1, the optimized formulation (F5) showed a vesicle size of 111.67 nm, an EE of 74.36%, a zeta potential of −7.253 mV, and a polydispersity index of 0.240. SA-transethosomes showed smooth and spherical surfaces as well as improved antioxidant activity and penetrability across the membrane as compared to control. Hence, it was concluded that SA-transethosome-based strategies have the potential to be useful for boosting the penetration of SA across the skin.

## Figures and Tables

**Table 1:** Composition of various transethosomes.

Formulations	PL90 (%, *w*/*w*)	SDC (%, *w*/*w*)	Ethanol (%, *v*/*v*)
F1	90	10	20
F2	90	10	30
F3	90	10	40
F4	85	15	20
F5	85	15	30
F6	85	15	40
F7	80	20	20
F8	80	20	30
F9	80	20	40

## Data Availability

Data is contained within the article.
